# Electrolyte imbalance in critically ill paediatric patients

**DOI:** 10.12669/pjms.35.4.286

**Published:** 2019

**Authors:** Faizia Naseem, Ayesha Saleem, Imtiaz Ahmed Mahar, Fehmina Arif

**Affiliations:** 1 Faizia Naseem, MBBS, FCPS. Assistant Professor, Paediatric Unit I, DUHS, Karachi, Pakistan; 2 Ayesha Saleem, MBBS, FCPS. Assistant Professor, Paediatric Unit III, DUHS, Karachi, Pakistan; 3 Imtiaz Ahmed Mahar, MBBS, DCH. Senior MO, Dr. Ruth KM Pfau Civil Hospital, Karachi, Pakistan; 4 Prof. Fehmina Arif, MBBS, FCPS. Paediatric Unit-I, DUHS, Karachi, Pakistan

**Keywords:** Critically ill children, Electrolyte imbalances, Morbidity, Mortality

## Abstract

**Objective:**

To determine the frequency and outcome of electrolyte imbalance in seriously ill children admitted in Paediatric Intensive Care Unit (PICU) of a public sector hospital in Karachi.

**Methods:**

All children between the ages of one month to 12 years admitted in PICU from May 2017 to October 2017 were included. Blood samples were drawn to determine the baseline sodium, potassium, calcium, magnesium and phosphorous and followed 24 hourly or earlier, if needed (Those who had imbalance at admission or on subsequent repeat labs as per protocol).

**Results:**

A total of 101 children were included in the study. Electrolyte imbalance was seen in 84%. A single electrolyte imbalance was noted in 30.58%. Hypocalcemia was the most frequent abnormality noted in 57.6%. Among the total expiries during the study period 91% had electrolyte imbalance. Mortality within 48 hours and duration of stay was also increased in these patients.

**Conclusion:**

Electrolyte imbalance is an important prognostic indicator in critically ill patients.

## INTRODUCTION

Electrolyte imbalances are not uncommon in critically ill paediatric patients.^1^ When present; they can significantly affect the outcome of patients. Critical care provision through paediatric intensive care units (PICU) is aimed at maintaining ‘homeostasis’ in the body which is vital for the organ’s support and optimal function. This involves not only fluids but also electrolytes balance.^2^

Major electrolytes important in this regard are sodium, potassium, calcium, magnesium and phosphorus.^3^ Their imbalance in either direction i.e. lower or higher than normal values can affect cellular processes, which can significantly affect morbidity and mortality.^4^ These imbalances also result in longer stay in hospitals^5^, thus adding significantly to the costs of management. Thus early recognition and intervention to correct these imbalances is essential to avoid poor outcome.^6^

Five possible mechanisms for the occurrence of electrolyte imbalance are the underlying disease process, end organ injury, fluid and electrolyte interventions, use of medications with potential of electrolyte derangements and application of critical care technology i.e. positive pressure ventilation.^7^

There is a lot of published adult data addressing this problem but paediatric data has just begun to generate especially over the last decade with the growth of paediatric critical care medicine subspecialty. This study was conducted to identify the magnitude and various aspects of the problem in Pakistani children admitted in Paediatric intensive care unit of a public hospital.

## METHODS

This was an observational study conducted over six months from May 2017 to October 2017, in Dr. Ruth KM Pfau Civil Hospital Karachi after approval from IRB (Institutional Review Board). Children admitted in PICU during the study period aged one month and older up to 12 years of age, were enrolled after written consent from their parents. Children with history of using electrolyte solutions prior to admission were excluded. All the enrolled patients were followed from admission till their stay in PICU. They were regularly monitored for the presence or later development of electrolyte imbalance. A detailed history and thorough physical examination was done. The demographic data of all the participants regarding age, sex and admitting diagnosis was noted.

Blood samples were drawn on admission to find out the initial serum levels of sodium, potassium, calcium, magnesium and phosphorus. There after the levels were repeated every twenty four hourly or earlier (if needed). Any value below or above the following cut off values was considered abnormal indicating electrolyte imbalance.^8^

**Sodium:** 135- 145 mg/dl

**Potassium:** one month to five month: 3.5-5.6 mg/dl

**Six month to one year:** 3.6-6.1 mg/dl

**>1 year:** 3.3-4.6 mg/dl

**Calcium (Total):** child: 8.8-10.8 mg/dl, Thereafter 8.4-10.2 mg/dl,

**(Ionized calcium):** 4.5-5.6 mg/dl.

**Phosphorus: 1-3 year:** 3.8-6.5 mg/dl

**4-11 year:** 3.7-5.6mg/dl

**> 12 year:** 2.9-5.4 mg/dl

**Magnesium:** up to 2 year: 1.6-2.6 mg/dl

**2-14 year:** 1.5- 2.3 mg/dl

Types of intravenous fluid given to patient, use of diuretics, steroids, inotropes and positive pressure ventilation were noted. Complications during stay like development of acute kidney injury (AKI), multi organ dysfunction syndrome (MODS), congestive cardiac failure (CCF), and syndrome of inappropriate secretion of ADH (SIADH) were noted. The final outcome i.e. discharges or expiry along with length of stay in PICU was also noted.

Data were analyzed using SPSS version 16. Descriptive statistics were applied to describe the results in terms of percentages and frequencies. Chi square was applied for association of electrolyte imbalance with the outcome, P-value < 0.05 was considered significant.

## RESULTS

During the study period, 101 children admitted in PICU were enrolled for the analysis. Among these children, 61 (60.39%) were male and 40 (39.60%) were female. Seventy five (74.25%) were under five years of age, 17 (16.8%) between 5 and 10 years of age and 9 (8.91) were above 10 years of age.

Regarding the admitting diagnosis, majority i.e. 31 (30.69%) had respiratory illness, 26 (25.7%) had neurological illness, 18 (19.80%) has sepsis/infection, 12 (11.88%) had cardiovascular problem, 4 (3.96%) had gastrointestinal ailment and 10 (9.90%) were categorized in miscellaneous group including endocrine diseases.

Electrolyte imbalance was noted in 85 (84.15%) children. It was present in 80 (94.11%) at the time of admission. Development of imbalance later during the stay was noted in 25 (29.41%) including 5 (5.88%) cases free of imbalance at admission (Table-I). Majority of them i.e. 26 (30.58%) had imbalance of a single electrolyte. However; 22 (25.88%) had imbalance of two electrlytes, 20 (23.52%) had imbalance of three electrolytes, 11 (12.94%) had imbalance of four electrolytes and 6 (7.05%) had imbalance of all five electrolytes (Table-I).

**Table I:** Descriptive characteristics of study population (N=101).

Characteristics	No	%
*Sex :*		
Male	61	60.39
Female	40	39.60
*Age groups:*		
< 5 years	75	74.25
5-10 years	17	16.83
>10 years	09	8.91
*Electrolyte Imbalance:*		
-Present	85	84.15
Absent	16	15.84
*Outcome:*		
-Discharged (including 2 LAMA and 2 referrals)	78	77.22
-Expired	23	22.77
*Disease Distribution:*		
-Respiratory	31	30.69
-CNS	26	25.74
-Infectons/Sepsis	18	17.82
-CVS	12	11.88
-GIT	04	3.96
-Others	10	9.90
*Length of stay in PICU:*		
<48 hours	42	41.58
48 hours–4 days	27	26.73
5 days-9 days	19	18.81
>10 days	13	12.87

The most frequently noted abnormality was hypocalcemia seen in 49 (57.64%) patients (Table-II). Hypernatremia was seen in 32 (37.64%) cases and hypophosphatemia was present in 30 (35.29%), Hypokalemia was noted in 26 (30.58%) and hypermagnesemia in 18 (21.17%) (Table-II). Regarding use of medications having potential for electrolyte imbalances, 75 (74.25%) required inotropes for stabilization, 61 (60.39%) required steroids and 44 (43.56%) needed diuretics. Mechanical ventilation was required in 56 (55.44%) children (Table-III). In-depth analysis of patients having electrolyte imbalance revealed that morbidity and mortality were both increased in such cases i.e. all AKI (Acute kidney injury), MODS (Multiorgan dysfunction syndrome), CCF (Congestive cardiac failure) and SIADH (Syndrome of inappropriate secretion of ADH) cases had electrolyte imbalance.

**Table II:** Pattern of electrolyte imbalance (n=85).

Electrolyte	No	%
*1. Sodium:*		
Hypernatremia	32	37.64
Hyponatremia	20	23.52
*2. Potassium:*		
Hyperkalemia	16	18.82
Hypokalemia	26	30.58
3. *Calcium:*		
Hypercalcemia	0	0
Hypocalcemia	49	57.64
*4. Magnesium:*		
Hypermagnesemia	18	21.17
Hypomagnesemia	06	7.05
*5. Phosphorus:*		
Hyperphosphatemia	10	11.76
Hypophosphatemia	30	35.29
Imbalance of single electrolyte	26	30.58
Imbalance of two electrolytes	22	25.88
Imbalance of three electrolytes	20	23.52
Imbalance of four electrolytes	11	12.94
Imbalance of five electrolytes	06	7.05
Imbalance on admission	80	94.11
Imbalance developed later	05	5.88

**Table III:** Morbidity profile and length of stay in PICU among patients with and without electrolyte imbalance.

Characteristics	With electrolyte imbalance (n=85)	Without electrolyte imbalance (n=16)	P-value
*Morbidity*			
*a. Required level of care:-*			
Need for ventilator (56)	46 (82.14%)	10 (17.85%)	0.08
Need for inotropes (66)	57 (86.36%)	09 (13.63%)	0.05
Need for diuretics (44)	40 (90.90%)	04 (9.09%)	0.08
Need for steroids (61)	50 (81.96%)	11 (18.03%)	0.08
*b. Complications:-*			
AKI	10 (11.76%)	0	0.29
MODS	11 (12.94%)	0	0.25
CCF	10 (11.76%)	0	0.29
SIADH	02 (2.35%)	0	0.79
*Length of Stay:-*			
< 48 hours	32 (37.64%)	10 (62.50%)	0.062
48 hours-4 days	24 (28.23%)	3 (18.75%)	0.42
5 days-9 days	17 (20.00%)	2 (12.50%)	0.54
>10 days	12 (14.11%)	1 (6.25%)	0.54
*Discharged:-*			
(Including 2 LAMA and 2 referrals)	64 (75.29%)	14 (87.50%)	<0.001

Out of these 101 admissions, total discharges from PICU were 78 (including 2 LAMA and two referrals) and there were 23 expiries. Out of the total expiries during the study period, 21 (91.30%) had electrolyte imbalance, making it a significant risk factor for mortality.

Electrolyte imbalance was also significantly seen (85.71%) in 12 out of 14 deaths in 1^st^ 48 hours of PICU stay. However the length of PICU stay was increased in each category of children with electrolyte imbalance especially beyond 48 hours.

## DISCUSSION

Among 101 enrolled children, overall electrolyte abnormalities were present in 85% (84.15%) of the cases. Since the five electrolytes were considered together and imbalance among any of them was noted, this might be the reason for the high incidence. Most of previous studies focused on only one or two electrolytes. Rao and Thomas, found the incidence to be 32%, but they focused only on sodium and potassium.^1^ Panda and Save mentioned 44.3%.^5^

Cummings BM found potassium abnormalities alone to be around 40%.^9^ Study done by Agarwal N, looking at all five electrolytes showed 60% incidence.^3^ This proves the statement (and rather modifies it) that electrolyte abnormalities are very common in critically ill children.

Majority had respiratory (30.69%), neurological (25.74%) and infective/sepsis (19.80%) etiology as noted in previous studies.^3^,^10^,^11^ The reason for less number of gastrointestinal cases (3.96%) could be the initial stabilization in ER and later shifting to ward upon improvement thus bypassing the PICU stay. This is important because gastroenteritis in children is the major source of electrolyte imbalance, but prompt and proper treatment may avoid PICU admission.

Although majority had abnormality of a single electrolyte, mixed disorders with combination of two, three, four and all five electrolytes were also seen (Table-II). Majority i.e. 80 (79.20%) had the abnormalities at the time of admission, 20 of them developed additional imbalances during stay. While five patients who were free of imbalance at admission developed them later, indicating the possible mechanism pointed earlier. Regarding the patterns of electrolyte imbalance, hypocalcemia was the most frequent abnormality noted in 49 (57.4%) cases (Table-II). Previous studies have mentioned the incidence of hypocalcemia in critically ill children to be around 40%^3^ and 47.5%.^12^ One possible explanation for such a high incidence of hypocalcemia is the high prevalence of Vitamin D deficiency in Pakistani children up to 77%.^13^

The presence of dysnatremias (either hypo or hypernatremia) in intensive care unit has been reported to be around 30%.^14^,^15^ Most of the literature has reported hyponatremia to be more prevalent than hypernatremia i.e. 23.2% vs. 16.7%^16^, 27.43% vs. 3.5%^5^, 50.5% vs. 9.4%.^3^ Sachdev A noted hyponatremia to be 19.3%.^11^

However, we found hypernatremia in 37.64% cases and hyponatremia in 23.52% cases. This might be due to the institutional policy of maintenance intravenous fluid to be the 0.9% saline rather than half strength or other hypotonic solutions in children above one month of age.

Hypokalemia was observed in 30.58% cases and hyperkalemia in18.82% cases. Previous studies have mentioned the incidence of hypokalemia as 40%^9^, 34.4%^3^ and 22.1%^16^ and hyperkalemia as 11.2%^16^, 16.12%^3^ and 29%.^9^ Hypophosphatemia was noted in 35.29% cases. This is similar to the incidence reported by Antachopoulos as 37.5%.^17^ Although ME Santana found the incidence up to 61% in critically ill children.^18^

As far as magnesium is concerned, we found more cases of hypermagnesemia up to 21% than hypomagnesemia in 7% (Table-II). This is in contrast to most of the published data showing hypomagnesemia to be more common than hypermagnesemia.^2^,^3^ In 17 out of 18 cases, it was seen as a part of mixed electrolyte disorders. Isolated hypermagnesemia was seen in a single case only.

Morbidity was significant in cases of electrolyte imbalance both in terms of required level of care and emergence of complications. Ventilatory support was required in 82% of these children. Similarly diuretics were required in 90.90%, inotropes were required in 88% and steroids were needed in 81.96%, indicating the need for high level of care (Table-III).

It is claimed that medications commonly used in intensive care units may contribute to the electrolyte disturbances as they can interfere with the absorption of electrolytes, alter hormonal responses affecting hemostasis and can directly affect the organ function as well. Their requirement however indicates the severity of illness and they continue to be an important risk factor for later development of electrolyte imbalance.^19^

Morbidity in terms of complications was also significant in such cases i.e. AKI, MODS, CCF, and SIADH were seen exclusively in patients having electrolyte imbalance (Table-III). Regarding the outcome, significant number of discharges were seen in cases without electrolyte Imbalance, P-value < 0.001 (Table-III). Overall mortality in our study was 22.77% (23/101), close to the mortality documented by Panda I as 23.73%^5^, and by Jan M et al as 22.8%^20^ (Table-IV). However, more than 90% of non survivors i.e. 21 out of 23 had electrolyte imbalance P-value <0.01 and among these 18 had imbalance at presentation P-value <0.001 (Table-IV) making the presence of electrolyte imbalance at admission to be the strongest predictor of mortality because such abnormalities complicate the course of illness, irrespective of primary disease process.^3^,^5^

**Table IV:** Mortality profile among patients with and without electrolyte imbalance.

Characteristics	With electrolyte imbalance (n=85)	Without electrolyte imbalance (n=16)	P-value
Expiries (23	21	02	<0.001
Within 48 hours (14)	12	02	0.001
-b/w 48 hours-4days	4	0	0.001
-b/w 5days-9days	3	0	0.005
>10 days	2	0	0.02
*With imbalance of:-*			
Up to two electrolytes	7	0	<0.001
Three or more electrolytes	14	0	
*With imbalance seen:-*			
On admission	18	0	<0.001
During stay	3	0	

Mortality during first 48 hours of stay was seen in 14 cases, among which 12 (85.71%) had electrolyte imbalance again pointing towards its significant role in poor outcome. Both single and mixed electrolyte disorders were noted and the mortality was increased in ascending order with the number of electrolyte involvement i.e. 7 of 21 non survivors had up to two abnormal electrolytes and 14 of 21 non survivors had three or more abnormal electrolytes (Table-IV).

Length of stay less than 48 hours was seen in 32 (37.64%) cases of electrolyte imbalance (high early mortality) whereas it was seen in 10 (62.50%) cases without imbalance (indicating high rates of recovery). However, the length of stay was increased in all other categories in patients with electrolyte imbalance (Table-III). This was consistent with studies conducted in the past.^5^,^11^,^12^ This makes electrolyte imbalance a major drain on limited health resources of poor and developing countries.

## CONCLUSION

Presence of electrolyte imbalance at the time of admission is an important prognostic indicator in critically ill children irrespective of primary disease process and needs to be addressed aggressively.
